# Integration of ethnic minorities during group-work for vocational teachers-in-training in health studies

**DOI:** 10.5116/ijme.5862.505a

**Published:** 2017-01-28

**Authors:** Ursula Småland Goth, Oddhild Bergsli, Else Marie Johanesen

**Affiliations:** 1 Faculty of Health, VID Specialized University, Norway; 2 Department of Vocational Teacher Education, Faculty of Education and International Studies, Oslo and Akershus University College, Norway

**Keywords:** Integration, intercultural communication, clinical practice, health professionals, group work, migrant, health care worker, Norway

## Abstract

**Objectives:**

To determine how to enhance integration of minority students in health education, and thereby improve intercultural communication skills and cultural sensitivity in a sample of health teacher students in Norway.

**Methods:**

After a group-work intervention and for a period of six months afterwards we followed an “action research” approach and observed 47 health teachers-in-training in their first year at the Oslo and Akershus University College during classroom interactions. Data were qualitative and comprised student self-reports and survey results along with observations from three teachers, the authors of the study. Data were analyzed using a constant comparative approach with opinion categorization and an open coding procedure, with separate analyses performed on observations from minority students, majority students, and teachers.

**Results:**

Both ethnic majority and minority students experienced an increase in intercultural knowledge and problem-solving ability after the experience of an early intervention in their first academic year of tertiary education. Students reacted favorably to the intervention and noted in class assessments both the challenges and rewards of overcoming cultural barriers. Teacher observation notes confirmed that early intervention led to an increase in interaction and cross-cultural engagement between minority and majority students compared to previous years’ classes without the intervention.

**Conclusions:**

Early classroom intervention to promote intercultural engagement can prevent clique formation along majority/minority lines. The method used here, tailored group assignments in ethnically diverse working groups at the very beginning of students’ tertiary academic career, can be an effective approach to cultivating attitudes and skills fostering intercultural awareness and sensitivity.

## Introduction

Over the last 40 years, European societies have become more and more diverse, with significant effects at all levels, including on students' needs and corresponding pedagogical approaches. Governments in Europe that once embraced a multicultural approach to diversity are now shifting emphasis to civic integration and assimilation.^1^ However the fact of increasingly culturally diverse societies means there can be no absolute return to education's traditional gearing and nationalizing of lifeworlds.^2^ Tertiary education, especially in the social sciences and in training for human service professions, expects self-conscious reflection on cultural differences from the students.^3^ Norway, in particular, has grown much more diverse in the last 25 years. Over 14% of Norway’s population is now immigrants, from 223 countries and autonomous regions.^4^ The capital, Oslo, has the country's highest proportion of residents with an immigrant background (32%).^4^ In 2015, approximately 10% of Norway’s higher-education students were immigrants.^5^ The educational system is, as always, a key interface for cultural integration. Particularly in pedagogy for health-related fields, domains where cultural sensitivity are vital for effective care, it falls significantly on vocational trainers for health-care and social-care students to transfer knowledge and cultivate the requisite skills.^6^^-^^8^ Immigrants – in their roles as students, co-workers, and patients – are confronted with stereotyping and prejudice, for which insularity and homogeneity among are often breeding grounds.^6^^-^^9^ Social-service providers are at risk for prejudicial attitudes if they have rarely engaged those who are different, confronted unfamiliar cultural boundaries, or cannot put themselves in the shoes of an immigrant struggling to understand the majority language. The studies just cited highlight intercultural challenges students face. In this article, we define minority and majority students not by ethnicity but by how “others” define the student and by that how students consider themselves.^8^ As well, “minority” or “the others” do not refer to homogeneous groups, but groups comprising individuals with potentially highly variable values and world views.^10^

In an educational setting, group work is regularly used as a didactic tool that challenges students to collaborate and integrate diverse skills and viewpoints.^11^ Diversity can be a mixed blessing for teams, potentially contributing to performance on the one hand while increasing potential for process loss, on the other.^12^ Diversity in a group often increases obstacles to internal coordination. One factor is prejudice, here defined as an unjustified negative attitude toward a person based solely on that individual’s membership in a group other than the majority. Still today immigrant students experience majority ethnocentrism, a tendency to regard one's own ethnic group, nation, religion, or culture as better or more correct than others.^7^ A study by Fischer and Derham^13^ showed that unfamiliarity of majority students with students with an ethnic (minority) background was associated with greater inter-group bias, making cultivation of familiarity, as Fischer and Derham suggest, a key intervention strategy. Potential benefits go beyond reducing prejudice, as diverse work groups tend to be more productive, provided they can successfully integrate their collective resources.^14^ Group work is especially valuable as a pedagogical tool in the health sector, where a society's diversity will inevitably be represented, and students need the skills to interact effectively and work together with patients and co-workers from a wide variety of backgrounds.

Even while diversity has become an essential concept in tertiary health-care education, minority students still meet obstacles to active classroom participation.^7^ In the Norwegian context, the authors have noted in their classes tendencies for minority students to be marginalized in class, with respect to participation, engagement (formal and informal) with fellow students, and even seating arrangements. Factors contributing to this situation include lack of skills to negotiate cultural differences, shyness, language difficulties, as well as stereotyping and prejudice. Over time, divisions can harden in a student cohort as social patterns become routinized and cliques sometimes form. We aimed to address these problems by designing an intervention at the very beginning of the students' tertiary academic career to foster collaboration and creative group problem-solving^14^ increasing student’s intercultural sensitivity and communicative skills.^6^^,^^7^ Can early intervention, at the very beginning of students' first academic year, further the necessary skills, attitudes, and habits?

The aim of the present study was to test a didactic approach as an intervention using “multi-ethnic group work” at the beginning of tertiary education to forestall the exclusion of minority students to enhance integration of minority students during health education.

## Method

The intervention was designed based on results gained in a pre-study two years earlier,^7^ which found that majority health students tried to avoid working with minority students, who, in turn, aimed by contrast to work in integrated groups. Throughout the work-group task and afterwards, we the teachers encouraged a multicultural-friendly climate in the classroom, with an emphasis on minority students not being marginalized. As the work groups proceeded, we looked for interaction and interchange of informal knowledge between majority- and minority students and the latter is full integration into group tasks.

### Study design

We followed an “action research” approach,^15^ in which lines are blurred between researchers and participants. The authors, simultaneously reporting and analyzing outcomes, hold teaching positions, and the key informants for the research were their students.^16^ We aimed only to gather qualitative data as a basis for both refinement of the technique and develop hypotheses for further quantitative research.

### Participants and setting

The study took place at the Oslo and Akershus University College, Faculty for Education and International Studies, and participants were first-year students in the study program “Vocational teacher trainer’s education for health- and social care”.  The classes consisted of 47 (43 female, four male) students, who shared the common goal of becoming vocational teachers in nursing, health work, or for childhood and youth services. In this group were seven minority students, originating from Turkey, Kurdistan (Turkey), Morocco, Iran, Bulgaria, and Pakistan. One of the minority students was Sami, an ethnic minority in Northern Scandinavia.

Our intervention occurred at the beginning of the term, before students in the class could form stable patterns of interaction. It was designed to be a group project serving to “invert” the expected majority/minority status and knowledge differentials that our experience teaching, and a pre-study, led us to expect would quickly develop. Students were divided into seven work groups, each consisting of six or seven majority students and one minority student. Students were informed they would receive a grade based on the success of their group as a whole. The assigned role given the minority students was to pose as patients of their own ethnic background, but with the limited languages skills and cultural knowledge of Norway that might be expected of a newly arrived refugee. The success of the group in meeting the challenge required finding the approach and language to decode the “patient's” medical problem that he or she could express only with great difficulty. This put the minority student in the privileged position of possessing the secret knowledge that the other students would have to decode. To meet this challenge, the group as a whole had to engage and cooperate, finding and putting to use formal and informal resources among themselves. The work-group task lasted one afternoon. Four months after the work groups completed their tasks, we followed up with the students for their individual reactions to the assignment. Over that time, we continued to make notes on degree of interaction among majority and minority students in class and if they stayed in their initial work groups during this time period.

The project was submitted for ethical approval and accepted by the Norwegian Social Science Data Service.

### Data collecting method

Data originated from observation notes taken during the period of the first semester by the researchers during work-group exercises, reflection notes written by the students after the initial group-task, and a literature search on the topic performed prior to the intervention.

At the beginning of the study the authors OB, EMJ, and USG observed the groups during their initial work. At the end of the students' first week, reflection notes were collected (however five of the 47 students did not submit their notes). Thereafter the authors OB and EMJ observed over a time period of four months all seven groups during group work sessions, with observations recorded in a personal log (field notes) and analyzed by the authors individually and thereafter compared and discussed.^17^

Additionally, a literature review for with keywords “integration”, “migrant student”, “group work”, “interaction” informal knowledge transfer”, and “health worker” was performed by the author USG using the electronic database Embase (via Ovid). While 350 articles were retrieved only twelve articles were found directly relevant. A cross-verification of the results from our three data sources, verified the results of the study.^17^

### Data analyses

For the purpose of analysis, data were thereafter analyzed with a constant comparative approach.^17^^-^^19^ By use of Opinion categorization, the collected material was organized by the author USG into categories according to themes before analysis. In an open coding procedure, statements and event items were identified from their substance. New observations were compared consecutively with previously collected data. Categories were developed and relationships between them were detected.

## Results

The qualitative content analysis was based on three tranches of written response notes of participants' experiences from the researchers', the minority students', and the majority students'.

### The researchers' experience

The researchers' experiences from the previous eight years teaching this course was that minority students and majority students seldom were found joining each other during group work, with minority students often described as “passive” or “restrained”. The researchers in their notes agree that the intervention largely succeeded. Six of the seven work-groups showed high levels of equality and cooperation, with only one group showing recurring signs of internal hostility based on intercultural conflict and ensuing defensiveness and dis-coordination. But for the considerable majority of the students, it appears that the work-group intervention at beginning of the semester helped establish, across the class's ethnic lines, the value of minority students' informal knowledge and their greater cultural fluency. We found further a tendency for minority students, once included as active members in a group with majority students at the start of the course, to remain in those groups. However, this was not always the case.

Field notes from teacher EMJ at the end of the observation period indicate that not all students stayed integrated within their original groups. Here the teacher EMJ wrote

“What I noticed was that two minority students entered the classroom while there were still several vacant seats at a group table with majority students, and yet they still chose to sit at a table without anyone else….  I was observant because we in previous years have heard that majority students did not want to include minority students in their group work because of their lacking language skills...” (Teacher EMJ, translated quote from Norwegian)

Even if not all minority students found a study group in which they felt closely associated, researchers' notes over the entire four-month study period show, compared to previous years' classes, improved cross-culture interaction in class and correspondingly improved learning outcomes on skills related to intercultural sensitivity and communication for both minority and majority students,

“...they [the group] solved the task in cooperation with each other. In the beginning, the Norwegians [majority students] explained the general tasks...” (Teacher OB, field notes on group 2, translated quote from Norwegian).

We find the group exercise sparking cultural curiosity on the part of majority students that is rarely seen in the class previously.

Questions addressed to the minority student,

“...What is usual in your home country? What do you eat – and what can it be substituted with here?"  (Teacher USG, field notes on group 2, translated quote from Norwegian)

Teachers' assessments of students’ learning outcomes are based on reflection notes and observation notes. The three teachers / researchers agree that in six of the seven work groups minority and majority students interacted as equals. We find a correlation between those work groups that achieved greatest internal equality between their majority and minority members and our assessments of which groups learned more. As well, we found a separate correlation between those groups highest in diversity (in terms of personal characteristics, age, and cultural background) and learning more.

A quote from a majority student during discussion in class exemplified this correlation:

 “...We in the student group X were surprised – we gained insight and increased our professional cultural skills. By interacting with immigrants, we saw their expertise. Here the understanding of cultural differences was particularly important for us. We learned so much”. (Teacher EMJ, translated field notes)

Based on students' individual reflection notes and our observation notes we saw, compared to previous years, an increase in intercultural knowledge and problem-solving ability.

### The minority students’ experience

Feedback notes from students and our observation notes showed that minority students felt appreciated for the active role they take in the work groups and the informal knowledge they share, leading to an experience of inclusion and integration into the class community.  In her reflection notes minority student writes,

 "It was good to talk about culture [differences], it tears down barriers.... Group work is a good exercise for us students. We hear about experiences and knowledge the others have. This is important for each and every one of us.” (Minority student no. 31, translated quote from Norwegian)

Closer collaboration with majority students via the work groups was credited by minority students as a means to improve proficiency in the Norwegian language, knowledge about unwritten cultural rules, as well as increasing their social capital, though these important goods don't always come easily.

Field notes taken by teacher USG quoting a minority student state,

“...ugh what a learning curve I experienced!” (Teacher USG, field notes quoting a minority student, translated quote from Norwegian)

Because the work-group task forced students to draw on each other’s' varied informal competences and specific know-how, the students' mutual exposure and engagement seemed enhanced, independent of their majority/minority status.

### The majority students’ experience

Majority students in their responses overwhelmingly responded favorably to the multi-ethnic work groups focused on addressing intercultural communication. The following is a typical sentiment,

“... I learned that there are various ways to reach a common goal...”. (Majority student no. 24, translated quote from Norwegian)

Our classroom experience from previous years was that majority students don't engage minority students, who in turn separate out into their own cliques, but the enforced interaction of the work-group intervention led to appreciation on the part of majority students for offering them a new learning experience. In this regard, a majority student said,

 “....I think it is an advantage that the training staff establish the various groups. In that way, we get to know each other better and to avoid ostracism …” (Majority student no. 21, translated quote from Norwegian).

Echoing that sentiment, another majority student wrote,

 “...Today I have learned about various cultures from different countries. Learned how important it is with diversity in the society and not to prejudge other people...” (Majority student no. 37, translated from Norwegian).

Majority student no. 21 said,

 “...I have learned a lot about cultural differences, and that there are actually not so large discrepancies where I expected them and larger differences where I did not expect them...” (Majority student no. 21, translated from Norwegian)

We found that many students reflected about their own reactions as they engaged fellow students with different cultural backgrounds.  The translated quotes from majority student no. 15, no. 35, and no. 38 illustrate this clearly:

“...Today I have learned that everybody has a different way of expressing themselves…and how important it is to try to understand others with a diverse background...” (Majority student no. 15)

 "What fantastic resources we have in our [minority] co-students, to get insight into the lives of others with a diverse background...” (Majority student no. 35)

 “...I have learned a lot about myself, how I react in a group with strong personalities, and I learned a lot about culture and diversity as well, as I got acquainted with the others [minority students] in the group...” (Majority student no. 38)

Both the students’ logs and observation data indicated that almost all the majority students expressed appreciation about the possibility to meet minority co-students with whom they normally would not have significantly engaged.   On the other hand, not all of the work-groups functioned smoothly all the time, misunderstandings, and conflicts relating both to personalities and to intercultural misunderstanding came up. Two majority students in one of the seven work-groups, group 2 in particular, were reluctant participants in the intervention already from the very beginning. This was noted in one teacher’s comment,

“...students don’t manage to utilize formal and informal competence..." (Teacher EMJ, field notes on group 2)

Here members of a group were diverted into conflict that arose, in the opinion of the observing teachers, from misinterpretations within the group of statement from members who intended to act cooperatively but were in some cases read otherwise. Even where cooperation broke down, the result was not necessarily a failure. From our teachers' perspective, those obstacles felt like points along a learning curve where majority students are facing internal cognitive barriers (such as stereotyping and prejudice) in coming to recognize that diversity exists and that it can be a valuable resource.

Even amidst this group’s difficulties, some of its participants reported valuable lessons. For example, a majority student said,

 “…actually it has been to our advantage that we can benefit from each other’s experiences and at the same time establish relationships with other students…” (Majority student no. 25, translated quote from Norwegian)

In general, we can say that we registered a high level of task cohesion in the groups, here especially here where students acted unified as teams to accomplish their assigned goals.    Teachers’ field notes and student feedback corroborated that early group work intervention could enhance integration of minority students during education and help improve communication skills and cultural sensitivity.

## Discussion

When diversity is managed well in a university setting and cultural differences are acknowledged and engaged, diverse classrooms in a diverse faculty contribute to the shaping and modeling of behaviors that encourage integration and cooperation.^20^ The cultural diversity of a group connects with its possessing different kinds of knowledge and expertise, which in teams or work groups tend to improve their outcomes, particularly on difficult tasks.^21^ One of the benefits of our classroom intervention was making this insight manifest to many of our majority students. As the individual students grade in the task we assigned is based on the groups’ achievement, and many students in Norway want to belong to groups ensuring them the highest grade.^7^ A frequent corollary to increased group diversity, increased process time for decision-making,^14^ also was manifested in our experience with the work-groups and was noted by students. If the group-task had been designed to be more time-constrained, this “downside” to diversity might have negatively impacted students' experience of the intervention.^21^^, ^^22^

While many of the majority students, we feel, require more knowledge and skills about cultural diversity in their curriculum,^7^ we felt that the intervention succeeded in that minority students generally experienced acceptance into the fabric of class life beyond simple tolerance. Both minority and majority students, we feel, developed into more independent learners through the group-work, gaining both intercultural understanding and insight into the unwritten rules of Norwegian society.

Relationships are a key source of one’s social capital.^21^^, ^^23^^, ^^24^ Perhaps because they are often socially disadvantaged, or perhaps reflecting cultural differences, minority students, research shows, place greater importance on the cognitive dimension of education and are less concerned with the social dimension.^7^^,^^25^ But the latter cannot be ignored, especially in the health professions and vocations significantly involving the arts of social interaction,^7^^,^^26^ and especially as cultural diversity becomes a fact of life in societies, such as Norway, with little prior experience. Integrating minority students into formal and informal engagement with their majority peers through group-building processes helps “expand the pie” of social capital for all, while particularly benefiting minority students who in the past were socially marginalized.^24^^,^^27^^,^^28^ We feel it is vital to undertake this task at the very beginning of students' academic career before social patterns and cliques become entrenched.^29^

We conclude that an early intervention in the students' first academic year of tertiary education enhances the learning process in classrooms with a mix of minority and majority students and provides a learning opportunity to develop intercultural communication skills. We recommend group-work task that draws on and privileges the informal knowledge of minority students, normally at a cultural disadvantage, both for furthering the integration process of the individual student, as well as for raising understanding, tolerance, cultural awareness, and the level of intercultural communication for all.

### Limitation and implications of findings

We approached this case study with an interpretive rather than positivist epistemological perspective, so the information gathered is not assumed to be independent of the time, place, and persons involved.^30^ Furthermore, while we compared our impressions from recent years of teaching the same course, without a formal control group external validity is weak.  On the other hand, this anecdotal case study, undertaken by seasoned practitioners and informed by previous research, could be a useful example for teachers seeking greater cultural greater inclusion in their classrooms.^30^

We the authors USG, EMJ, and OB hold dual roles as teachers for the participating students and researchers for this study. We aimed always to self-consciously avoid bias and maintain the highest ethical standards,^17^ but it remains that students depended for their grades on their teachers' assessments. These factors inevitably represent a challenge to our findings.

A larger study of this intervention with a control group, a larger set of teachers implementing the technique, and longer follow-up would help better establish whether there is empirical warrant for using this technique more widely.

### Acknowledgements

The authors would like to thank the participants of the survey and the anonymous peer reviewer for their valuable advices and recommendations. USG is the main author and the corresponding author of this article. USG initiated the project, was responsible for the ethical approval, performed and analyzed the literature search and supervised the data collection. The authors of the paper (OB, USG & EMJ) collected the data and participated in the analysis. All authors participated in the writing process while USG was responsible for the layout and writing the final submitted version.

### Conflicts of interest

The authors declare that they have no conflict of interest.
